# Neurobiology of local and intercellular BDNF signaling

**DOI:** 10.1007/s00424-017-1964-4

**Published:** 2017-03-09

**Authors:** Manju Sasi, Beatrice Vignoli, Marco Canossa, Robert Blum

**Affiliations:** 1 Institute of Clinical Neurobiology, University Hospital, University of Würzburg, 97078 Würzburg, Germany; 2 Centre for Integrative Biology (CIBIO), University of Trento, 38123 Povo, TN Italy; 3 European Brain Research Institute (EBRI) “Rita Levi-Montalcini”, 00143 Rome, Italy

**Keywords:** Signaling, Synaptic localization, Synaptic plasticity, Long-term potentiation, BDNF, TrkB, Anxiety disorders

## Abstract

Brain-derived neurotrophic factor (BDNF) is a member of the neurotrophin family of secreted proteins. Signaling cascades induced by BDNF and its receptor, the receptor tyrosine kinase TrkB, link neuronal growth and differentiation with synaptic plasticity. For this reason, interference with BDNF signaling has emerged as a promising strategy for potential treatments in psychiatric and neurological disorders. In many brain circuits, synaptically released BDNF is essential for structural and functional long-term potentiation, two prototypical cellular models of learning and memory formation. Recent studies have revealed an unexpected complexity in the synaptic communication of mature BDNF and its precursor proBDNF, not only between local pre- and postsynaptic neuronal targets but also with participation of glial cells. Here, we consider recent findings on local actions of the BDNF family of ligands at the synapse and discuss converging lines of evidence which emerge from per se conflicting results.

## Introduction

**Fig. 1:**
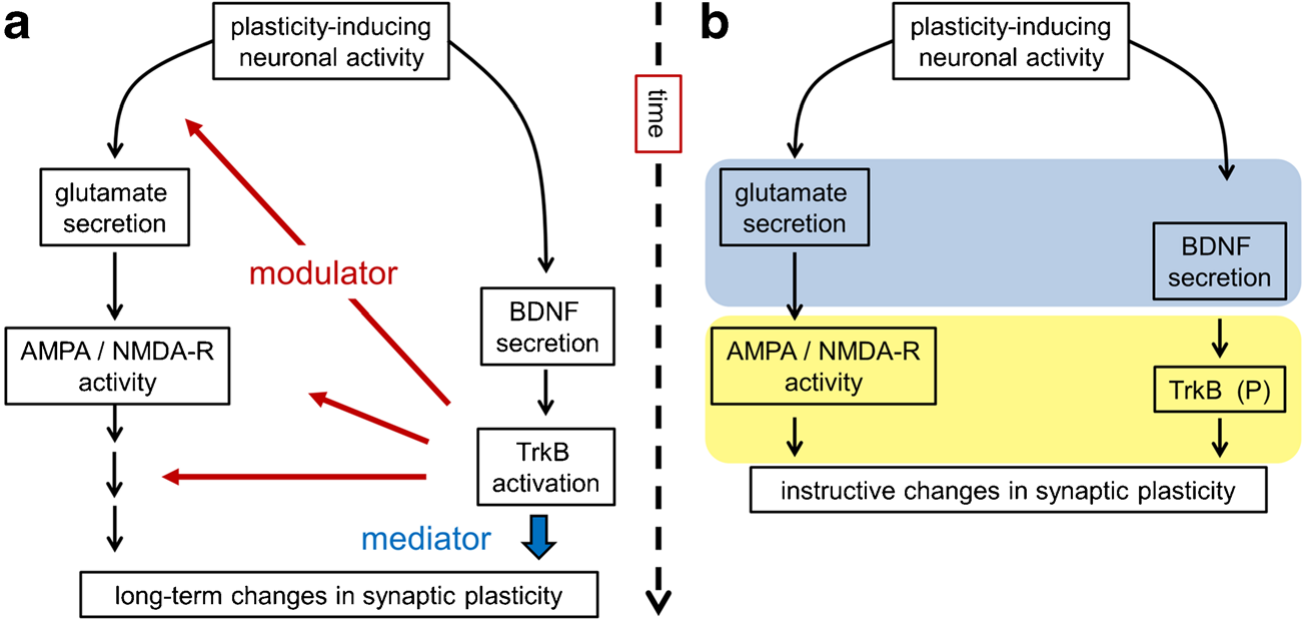
Overview of BDNF signaling. A major source of BDNF in the brain is the excitatory glutamatergic synapse, a principle synapse of synaptic plasticity, learning, and memory. During plasticity-inducing neuronal activity, BDNF and glutamate are released at synapses. **a** BDNF secretion occurs at a slower timescale than glutamate release. BDNF binds to its receptor TrkB to activate modulatory signaling cascades (see Fig. 3). In presynapses, BDNF-TrkB signaling enhances neurotransmitter release. On postsynaptic sides, BDNF/TrkB signaling increases the function or open probability of ionotropic glutamate receptors. Furthermore, it modulates signaling cascades downstream of neuronal excitation. BDNF as a mediator (according to Park and Poo [126]) can directly influence late effects in synaptic plasticity, for instance, local protein synthesis, spine remodeling, or gene transcription. **b** BDNF as an instructor of synaptic plasticity. Glutamate and BDNF are released within a critical time window (*bright blue*) and TrkB activation by BDNF serves as an instructive signal for associative postsynaptic long-term potentiation (*bright yellow window*)

On a neurobiological level, learning and memory depend on regulated signaling processes at synapses and involve the precise synaptic communication between neurons and other cellular partners. The neurotrophin brain-derived neurotrophic factor (BDNF) [15, 151] and its signaling partners [21, 30, 110, 126] have emerged as key regulators of synaptic plasticity, a biological process describing the regulation of synaptic strength by neuronal activity. Many neuromodulatory factors affect neuronal plasticity, but in contrast to many other factors involved in synapse function, BDNF may serve as a real mediator rather than simply a modulator of synaptic plasticity and synaptic communication [126] (Fig. 1a). Furthermore, BDNF and neurotransmitter signaling cascades can act together in a close temporal association to show immediate and instructive functions on synaptic plasticity (Fig. 1b). Therefore, much attention has been given to BDNF because specific interference with BDNF-related signaling is regarded as a leading strategy to stimulate neuronal and synaptic plasticity for potential protective and functionally restorative treatments for neurological and psychiatric disorders [97, 116, 152]. This review focuses on recent findings on the neurobiology of BDNF and how it acts in synaptic signaling. We conclude with some brief comments on how fundamental research on BDNF signaling can contribute to a better understanding of dysfunctional synaptic plasticity or disadvantageous synaptic learning in neuropsychiatric diseases.

## Neurobiology of BDNF

Brain-derived neurotrophic factor (BDNF) belongs to the protein family of neurotrophins, of which other members are the homologs nerve growth factor (NGF), neurotrophin 3 (NT-3), and NT-4 [90, 151]. BDNF is the predominant member of the neurotrophin family in the adult brain. Initially, BDNF was purified from pig brain tissue and was found to support survival and differentiation of sensory neurons [15]. Disruption of the *Bdnf* gene in mice causes the loss of certain types of neurons in the periphery but does not result in dramatic changes in the central nervous system (CNS) [48, 151]. These early experiments already indicated that the fundamental functions of BDNF and its receptor TrkB can differ widely between the peripheral and the central nervous system.

Human, rat, and mouse BDNF are expressed from a single gene locus, and *Bdnf* gene transcription is tightly regulated, cell-type specific, and controlled by neural activity. The structure of the *Bdnf* gene is highly conserved throughout mammals, indicating that there is a strong pressure on the conservation of the regulatory non-coding elements. The mouse and rat *Bdnf* genes consist of eight 5′-untranslated exons and one protein coding 3′-exon [3]. In humans, a latest evaluation found bidirectional transcription of the *BDNF* gene and total number of nine BDNF 5′ UTR promoters (termed I–IX. Please note that the present BDNF promoter nomenclature was claimed in 2007 [132]). A recently introduced genome editing approach combines an inactive Cas9 with a methylation or demethylation activity to specifically alter the methylation pattern of a gene, thus allowing research on causal relationships between changes in methylation and gene function [95]. Notably, targeted demethylation of the *Bdnf* promoter IV in post-mitotic neurons was sufficient to activate BDNF expression. This indicates that active, signaling-driven demethylation of a specific *Bdnf* promoter site contributes to activity-dependent BDNF activation [95]. The complex structure and regulation of *Bdnf* gene activity offers a broad susceptibility for regulated epigenetic control of *Bdnf* expression [28]. One can expect that activity-dependent and cell-type specific epigenetic regulation of the *BDNF* gene will become an influential topic in gene-environment interaction research (G × E) in humans.

Removal of BDNF from all neurons markedly reduces BDNF levels in the brain [136]. However, even after global neuronal deprivation of BDNF, some BDNF is still found in cortical tissues [136]. In line with this, microglial cells have been found to be another physiological source of BDNF [128, 154]. Microglial BDNF supports learning-dependent spine formation in the motor cortex, and BDNF removal from microglia reduces the performance in some motor learning tasks and other learning paradigms [128].

Currently, we do not know much about BDNF protein sources in humans. For instance, human platelets carry high amounts of BDNF, while mouse platelets lack BDNF. Human serum also contains a substantial amount of BDNF, while it is barely detectable (if not absent) in mouse serum. BDNF is undetectable in mouse megakaryocytes but highly expressed and stored in human megakaryocytes [29]. This suggests that alterations of BDNF levels in human serum, as reported in studies dealing with psychiatric diseases, might reflect changes occurring in megakaryocytes and platelets [29]. These data remind us that it is important to understand whether and how BDNF sources from outside the brain contribute to diverse bodily functions and brain plasticity.

In the peripheral nervous system, BDNF has the ability to prevent regulated cell death [14, 146]. In the brain, BDNF is not primarily acting as a survival factor but rather mediates region-specific effects on synaptic function and neuronal morphology [94, 120, 136]. For instance, loss of striatal BDNF signaling causes a spinal atrophy caused by defects in the dendritic complexity of GABAergic striatal medium spiny neurons [94, 136]. GABAergic striatal neurons do not produce BDNF but get significant amounts of axonal BDNF from presynapses of corticostriatal projections [4, 127]. The specific depletion of BDNF from corresponding axonal projections abolishes LTP at corticostriatal synapses [127]. In Huntington’s disease, an autosomal dominant neurodegenerative disorder, disturbed anterograde axonal transport of BDNF to the striatum causes reduced cortical supply of BDNF which is thought to be responsible for striatal degeneration [52]. However, in a Huntington’s disease model, defects in corticostriatal plasticity are caused by reduced engagement of the postsynaptic BDNF receptor TrkB. This suggests that defects in synaptic plasticity do not depend on cortical delivery of BDNF on striatal spiny projection neurons, at least early in Huntington’s disease [131]. It has recently been shown in another disease model that reducing mutant Huntingtin levels is sufficient to improve the supply of cortical BDNF on striatal neurons and to rescue the trophic defects [173]. These data might point to a relevant role of TrkB transactivation in striatal neurons, in which dopamine D1 receptor and BDNF/TrkB signaling are intertwined to modulate BDNF responsiveness [67]. This is in accordance with a cell-autonomous role of TrkB in striatal neurons [94].

High levels of BDNF messenger RNA (mRNA) and protein are found in the hippocampus [37, 136], a brain region of importance in contextual and spatial learning and explicit memory. In the hippocampus, neuronal anatomy of glutamatergic neurons shows only minor changes when BDNF is removed either from all cells [76] or selectively from all neurons [136]. However, this does not exclude the possibility that less abundant cell types, such as certain types of GABAergic neurons, are under developmental control of BDNF. In summary, these data show that axonal BDNF can dramatically influence the neuronal complexity of some specific types of neurons, but not all types of neurons.

## Family of bioactive BDNF ligands

New data revealed that three functionally different proteins originate from the *Bdnf* gene, namely the precursor proBDNF, mature BDNF, commonly termed as BDNF, and even the prodomain of BDNF [62]. The neurobiology of all three BDNF ligands is one of the most fascinating topics in BDNF research. Now, it is clear that all these BDNF-derived proteins are bioactive. However, there is an ongoing (and passionate) debate about the cellular locus where the conversion of proBDNF to BDNF takes place, which enzymes are responsible for the cleavage, how efficient this processing is, and how much and where proBDNF is secreted by neurons [7, 24, 43, 89, 103, 118, 122, 168].

BDNF carries an ER translocation signal peptide (pre-proBDNF). The signal peptide is cleaved off during import into the secretory protein transport pathway. The resulting proBDNF is further processed, and the propeptide is finally cleaved off to generate the mature neurotrophin of ~13 kDa. Mature BDNF forms stable homodimers that are secreted in both constitutive and regulated pathways. At certain central synapses or under certain experimental conditions, proBDNF is also secreted [17, 86, 103, 155, 165, 168]. Following secretion, proBDNF can be cleaved by extracellular proteases, leading to local formation of mature BDNF to instruct long-term changes in synaptic plasticity [122]. For a long time, the fate of the remaining prodomain of BDNF was unknown and believed to undergo rapid degradation. However, the prodomain is a detectable protein [7, 43] and undergoes activity-dependent secretion from hippocampal neurons [7].

There is compelling evidence that proBDNF and BDNF utilize distinct receptors to mediate opposing neuronal actions to regulate neuronal excitability, neuronal remodeling, synaptic communication, and plasticity in the CNS. While BDNF has the ability to increase neuronal excitability and synaptic strength [5, 20, 71, 76, 78, 130, 171], proBDNF can reduce neuronal excitability, decrease synaptic efficiency, and facilitate synaptic depression [53, 98, 126, 162, 165, 167].

## The human BDNF variants Val66Met

The prodomain of BDNF is the locus of a functional human BDNF polymorphism known to affect synaptic plasticity, learning, and memory processing, as observed with the help of functional magnetic resonance imaging [47]. This single nucleotide polymorphism, the so-called Val66Met polymorphism, is defined by replacement of valine66 with methionine (reference SNP rs6265). Memory impairments in Met66 allele carriers cause a higher susceptibility to neuropsychiatric disorders, perhaps because associative memory processing in the emotional circuit is also affected. Polymorphism frequencies vary between ethnicities and range from about 20 to 50% between populations. The Val66Met polymorphism does not exist in the mouse or other model organisms, and multiple studies are aimed at mimicking the function of BDNF^Val66Met^ with the help of cellular models, genetic tools, or genetically engineered mouse models [26, 33, 34, 47, 64]. In neurons, proBDNF with Met66 shows abnormal trafficking and an altered intracellular distribution. Interestingly, when both BDNF variants were expressed in the same neuronal cell, 70% of the proBDNF heterodimers carried one BDNF (Val) version and one BDNF (Met) version [34]. These dimers were found to be inefficiently sorted into secretory granules, which led to a decreased number of secreted BDNF proteins [34]. It is thought that perturbations in BDNF trafficking impairs CNS function and mood regulation because of an inefficient activity-dependent secretion of BDNF at synapses [33, 147]. However, reduced secretion seems not to be the only mechanism by which Met66 affects BDNF function. Met at position 66 induces massive structural changes in the prodomain and affects the biological potency of prodomain signaling. Met66, but not Val66, BDNF prodomain can induce growth cone retraction in young hippocampal neurons, an effect which includes signaling through the so-called sortilin-related VPS10 domain containing receptor 2 (SorCS2) [7, 62]. Not much is known yet, but the ligand functions of the Met66-prodomain might be responsible for certain human-specific features of neural plasticity.

In a large non-clinical sample of older men and women (41–80 years old), no association between the BDNF Val66Met polymorphism and changes in the mood status was found [149]. However, it needs to be noted that BDNF protein levels can change dramatically during development [75] and aging [59], which might explain this finding. For instance, BDNF modulates hippocampal aging [142], but it is not well known yet how aging interacts with BDNF function in synaptic plasticity, learning, and memory. It will be necessary to gather more information about the changes in BDNF levels and localization over the entire live span of model organisms and humans.

## Cellular aspects of BDNF secretion

There is an ongoing debate whether BDNF is primarily acting from the postsynaptic or presynaptic site (for review [21, 46, 126]). Conceptually, anterograde release refers to data showing that BDNF is synthesized in the somatic cell body area of the presynaptic cell, is anterogradely transported to synaptic or extrasynaptic structures, and is stored in dense core vesicles. In this model, secretion occurs from presynapses and is activity-dependent and regulated by calcium ions. The concept of postsynaptic secretion includes transport of BDNF encoding mRNA or BDNF protein to dendritic structures followed by activity-dependent release by specialized postsynaptic or endosome-like vesicles. Postsynaptic secretion of BDNF includes the possibility that BDNF is released from the presynaptic or postsynaptic cell, taken up by postsynaptic neuronal structures and is finally re-offered to the synapse via endocytic pathways. Furthermore, it has been recently shown that glial cells can recycle BDNF to re-secret it to neurons. There is experimental proof for all these concepts, and, as discussed later, there is no universally valid hypothesis that can explain the diversity of physiological BDNF actions.

Attempts to define the locus of functional BDNF secretion were complicated by the low amounts of endogenous BDNF normally found in neurons in vivo [43, 126]. Thus, neuronal cultures and genetic overexpression of BDNF in slice cultures became a standard tool to study BDNF synthesis, synaptic steady-state localization, and synaptic BDNF release mechanisms. In conclusion, these studies show that BDNF is stored and released from both axonal and dendritic compartments [12, 19, 24, 25, 45, 56, 57, 80, 103, 144, 164, 168]. Furthermore, BDNF becomes recycled for activity-dependent secretion [144, 164], and the recycled BDNF can then support activity-dependent changes in synaptic strength [17, 155]. At postsynaptic sites, recycling of exogenous BDNF occurs through endocytic trafficking pathways and involves the function of the specific synaptotagmin isoform 6, which is distinct from those involved in secretion from dense core vesicles [164]. This indicates that synaptotagmin isoforms might serve as useful markers to follow the fate of stored and released BDNF. Studies using neuronal cultures in vitro and overexpressed BDNF fused to the fluorescent proteins support the view that postsynaptic secretory granules in dendrites and postsynaptic spines are a preferential site of BDNF secretion [24, 45, 57, 80, 89].

**Fig. 2:**
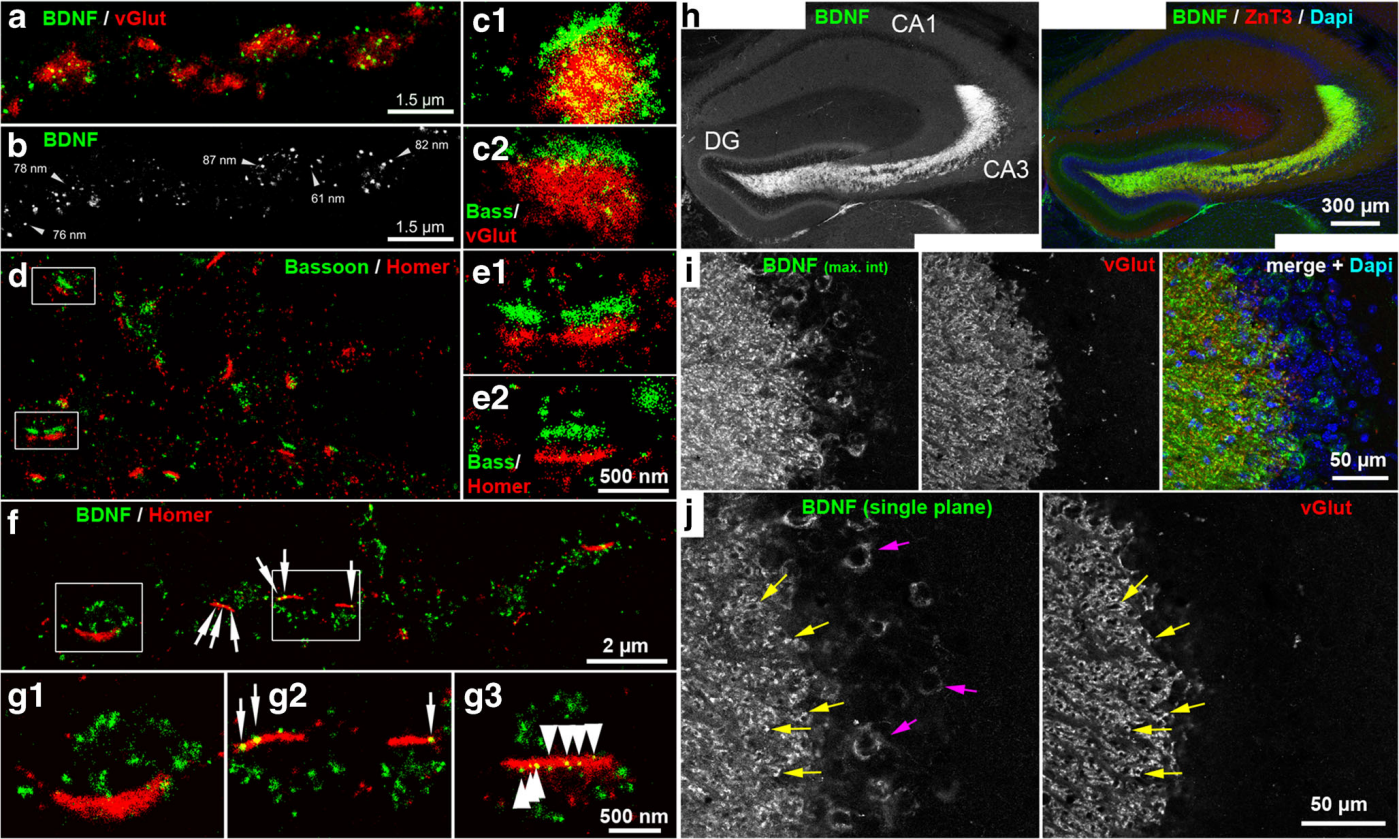
Immunodetection of BDNF in cultured hippocampal neurons and in the hippocampus of the mouse. **a**, **b** *d*STORM super-resolution images with a resolution of ~20 nm. Immunoreactivity of BDNF and presynaptic vesicular glutamate transporter (vGluT) is shown. Single BDNF-containing granules are located within the vGlut + area, representing the glutamatergic presynapse. **b** *Black-white* presentation of the BDNF granules from (**a**). Single vesicles with a dense BDNF label are pointed out by *arrows*. These granules have a diameter in the range of 60–90 nm. **c**_*1,2*_ (*details*) Presynaptic Bassoon bar structures and vGlut + disks. **d** Presynaptic Bassoon and postsynaptic Homer1 clusters shown as juxtaposed synaptic bar structures (mouse hippocampal neurons cultured for 35 days). **e**_*1,2*_ Homer and Bassoon form a bar-like synaptic scaffold structure. **f**, **g** BDNF localization at postsynaptic bar structures (DIV 30). BDNF+ vesicles accumulate in juxtaposed position to Homer + postsynaptic bars (white arrows). **g**_*1–3*_ Some BDNF + granules overlap with the postsynaptic bars. **g**_*3*_ Multiple (nine) small BDNF + vesicles are aligned within a postsynaptic, Homer + bar (*arrows*) (**a–g** *: taken from Andreska* et al. [8]). **h** Anti-BDNF immunoreactivity in the hippocampus of the mouse (8-week old, confocal microscopy). Note the overlap with ZnT3 (zinc transporter 3), a protein with high abundance in presynaptic mossy fiber terminals. DAPI labels cell nuclei. **i**, **j** BDNF immunoreactivity is pronounced in vGlut + mossy fiber terminals and in somatic areas of CA3 pyramidal neurons. **i** Maximum intensity projection; **j** single confocal plane (**h–j** *: performed by M. S. & R.B.*)

New techniques, mouse models, and rigorously specified anti-BDNF antibodies offered new insights in the steady-state localization of endogenous, natural BDNF and proBDNF in the mouse brain. In the BDNF-rich hippocampal circuit, BDNF is highly abundant in presynaptic terminals of glutamatergic neurons, where it is stored in presynaptic dense core vesicles. However, it was not found in distal dendrites, thus suggesting that BDNF exerts its synaptic action primarily in an anterograde fashion [43]. BDNF is highly abundant in the mossy fiber terminal, a large bouton synapse formed between granule neurons and CA3 pyramidal neurons [37, 38, 43, 168] (Fig. 2). In the same presynaptic structure, anti-prodomain immunoreactivity is observed [43, 168]. Whether uncleaved or cleaved forms of BDNF are stored in the mossy fiber terminals seems to be under developmental control. ProBDNF abundance and secretion might be pronounced during early and late postnatal development, when axonal extension and synaptic maturation are prevalent [168]. Indeed, it has been shown that refinement of functional connectivity in CA3 needs proBDNF/p75^NTR^ signaling [162]. In another large bouton synapse, the mossy fiber boutons on cerebellar granule neurons, high amounts of BDNF are stored as well. This axonal BDNF is functionally important, as it regulates the maturation of GABAergic synapses [31].

A preferential presynaptic storage of endogenous BDNF has also been visualized in long-term cultured hippocampal neurons, which develop hallmarks of mature synapses, such as pre- and postsynaptic bar structures (Fig. 2) [8]. Using direct STORM (dSTORM), a super-resolution fluorescence imaging approach enabling spatial lateral resolutions of ~20 nm, it has been shown that natural BDNF is located in granules directly within the fine structure of the glutamatergic presynapse [8]. A quantitative estimate confirmed that individual glutamatergic presynapses carried up to 90% of the synaptic steady-state BDNF immunoreactivity. A minor fraction of BDNF molecules was localized in small vesicles on postsynaptic bar structures. The BDNF content varied strongly between individual presynaptic varicosities. These data confirm that neurons are able to enrich and store high amounts of BDNF in small granules within the mature glutamatergic presynapse, at a principle site of synaptic communication. However, the study also showed that BDNF is not located directly in the active zone (Fig. 2), thus raising the question whether presynaptic BDNF is released from the active zone or from lateral parts of the presynapse [8].

BDNF transcripts carry two different 3′-untranslated region (UTR) isoforms, a long isoform and a short isoform. It has been described that the short 3′UTR BDNF isoform is a somatic isoform, while the long isoform is targeted to dendrites by dendritic mRNA transport mechanisms to be used for local protein translation, trafficking, and secretion [6, 36]. Using various quantitative techniques such as deep RNA sequencing, high-resolution transcript localization, quantitative PCR techniques, and 3′-end sequencing, BDNF mRNA was primarily found in the somatic compartment but was barely detected in dendrites. The vast amount of BDNF transcripts contained the short isoform, thus indicating that BDNF protein translation preferentially occurs in somatic regions of neurons [160]. It is not well understood yet how neuronal BDNF reaches its postsynaptic release structures to mediate autocrine functions on postsynaptic spines [45, 57, 157].

## Neuronal activity and BDNF secretion

In a simplified view, once established, BDNF expression and regulated secretion are under control of neuronal activity and depend on an increase in intracellular calcium at the BDNF release site, e.g., by activity-dependent calcium influx, the action of cAMP, or by the action of BDNF itself [12, 24, 25, 35, 54, 56, 57, 74, 80, 103, 144, 150, 168, 169]. Multiple signaling cascades for BDNF or neurotrophin secretion are experimentally verified and discussed elsewhere [21, 46, 126].

After secretion, BDNF acts as a local factor. Its biochemical characteristics prevent a broad diffusion within the target region. BDNF is a sticky protein of about 27 kDa (mature BDNF dimer) and is positively charged under physiological conditions. The isoelectric point of BDNF is close to ten [87, 130]. For this reason, BDNF is only locally acting at synapses [63, 78, 82, 161]. Local release of BDNF affects synaptic plasticity on an exquisitely local scale in the low micrometer range. For instance, when dendrites or cell bodies of a donor neuron supplied overexpressed BDNF to a nearby dendrite of a recipient neuron, the BDNF source had to be within a distance of 4.5 μm to induce dendritic growth in the recipient neuron [63]. Structural plasticity mediated by BDNF can even be restricted to a single spine [60]. The local action of BDNF in combination with its activity-dependent secretion opens the possibility that BDNF is released as a matter of coincidence between presynaptic and postsynaptic activity, meaning that BDNF release occurs at a local place, as a response to the synchrony of a local synaptic input and a postsynaptic spike. Indeed, pairing of repetitive postsynaptic spikes with local glutamate uncaging, a method to simulate glutamate release from presynaptic sources, induces local structural plasticity in postsynaptic spines [150]. This structural plasticity is represented by an immediate and a gradual phase of spine enlargement [150]. The gradual slow phase is dependent on protein synthesis and local BDNF secretion, which is induced by spike-timing plasticity [150]. In conclusion, BDNF may act as a synapse-specific, local actor for synaptic remodeling in associative learning events and is needed for the consolidation of efficient synaptic communication. Recent data underlined the impact of postsynaptic BDNF secretion for spike-timing-dependent plasticity in CA1 [45, 57, 60].

The timescale of secretion of natural BDNF is not well defined. Modeling BDNF secretion with overexpressed recombinant BDNF coupled to the green fluorescent protein GFP enables the direct visualization of the release of BDNF-GFP. Initial studies indicated that activity-dependent BDNF release is quite slow and occurs over seconds to minutes [24, 56, 80]. In contrast, physiological answers of exogenously applied BDNF may be very fast and can occur in the millisecond to second range [20, 21, 71, 79, 82, 91]. This is no discrepancy because BDNF binds with high affinity to its target receptor TrkB [138] and even low amounts of secreted BDNF should be able to exert a prominent action. A glutamatergic synapse carries multiple BDNF-containing granules distributed over the complete presynaptic structure [8] (Fig. 2). Therefore, secretion characteristics of BDNF coupled to a fluorescent protein observed by time-lapse imaging microscopy are likely to reflect the continuous but stepwise secretion of individual BDNF molecules from individual secretory structures. Indeed, when postsynaptic BDNF secretion was investigated with BDNF fused to superecliptic pHluorin, fast imaging visualized the release of BDNF in the range of milliseconds to seconds [57, 60]. Spike-like fluorescence signals representing BDNF release from postsynaptic sites correlated with local glutamate uncaging and fast and stepwise BDNF release depended on activity [57, 60]. Even glial cells, which take up synaptically released proBDNF from neurons, are able to rapidly re-release BDNF [17]. In conclusion, data looking at the speed of BDNF release with fluorescent proteins are in accordance with fast and slow BDNF effects on neuronal excitation and synaptic plasticity.

## The BDNF receptors TrkB and p75^NTR^: functional antagonism of neurotrophin signaling

The neurotrophins act by binding to two kinds of plasma membrane receptors, the tropomyosin (t)-receptor (r)-kinase (k) Trk [102] and the p75 neurotrophin receptor (p75^NTR^) [41]. BDNF (mature dimeric BDNF) binds with high affinity (dissociation constant ~10^−11^ M) to its receptor TrkB [138]. The binding of BDNF to TrkB has proven to be of elementary importance for the effects of BDNF to promote synaptic efficiency and long-term potentiation [2, 16, 18, 49, 51, 57, 76, 111, 112, 122, 130, 171] (Fig. 4a). Under physiological conditions, the dimeric BDNF can also act through p75^NTR^, but with much lower affinity (dissociation constant ~10^−9^ M). The preferential receptor for immediate proBDNF effects is p75^NTR^ [53, 69, 84, 155, 162, 165, 167], and p75^NTR^ and TrkB are the key players in the concept of functional antagonism of the BDNF signaling system [76, 111, 139, 170] (Fig. 4a). For instance, low-frequency stimulation of neurons can cause proBDNF release at excitatory synapses, which can consequently lead to an attenuation in synaptic function (long-term depression) via p75^NTR^ [118, 122, 167, 168].

A remarkable form of plasticity through proBDNF/p75^NTR^ was observed in the developing cortex and hippocampus [162]. Here, spontaneous activity of neighboring synapses was found to strengthen the synaptic connection. In contrast, neighboring synapses that were losing synchronicity and were only rarely co-active became depressed. Notably, when proBDNF was given to highly synchronized synapses, these local synapses underwent synaptic depression. This “out of sync – lose your link” plasticity mechanism points to a relevant role of proBDNF/p75^NTR^ signaling in activity-dependent shaping of synaptic connectivity [162].

TrkB, the concomitant BDNF receptor, is expressed from a single gene but exists in four versions, the full-length receptor tyrosine kinase TrkB, and the splice variants, the truncated version TrkB-T1 (glycoprotein 95), TrkB-T2 [108], and TrkB-T4 [50]. An isoform designated TrkB-T3 has been found in chicken. The precise function of truncated TrkB receptors still remains elusive, and it is not known yet whether the transcripts encoding for TrkB-T2 and T4 are translated in vivo to form functional proteins. However, the different TrkB transcripts are differentially regulated in phases of experience-dependent plasticity, e.g., in the visual cortex, [22], indicating that they might be used to interfere with TrkB expression.

BDNF-dependent effects on synaptic plasticity are generally mediated by the TrkB kinase, while the role of the truncated TrkB-T1 is not well understood. TrkB-T1 binds to BDNF with similar affinity as the TrkB kinase and can interfere with BDNF-TrkB signaling by binding BDNF without activation of the downstream kinase cascades. Overexpression in neurons or the knockout of TrkB-T1 from all cells affects the dendritic complexity of certain types of neurons, for instance, CA1 pyramidal neurons, dentate gyrus granule neurons in the hippocampus, or neurons in the basolateral amygdala [27, 107]. When overexpressed in neurons, TrkB-T1 affects structural and functional plasticity [107]. Furthermore, it reduces fast physiological answers of the TrkB kinase [140] and in vivo reduction of TrkB signaling by removal of one BDNF allele can be partially rescued by TrkB.T1 deletion. These studies indicate that there is a physiological interaction between TrkB and endogenous TrkB-T1, but neither the underlying signaling mechanism nor the function are known and it is not solved yet whether effects of endogenous TrkB-T1 are of neuronal origin. Fast signaling via the truncated TrkB-T1 has been found in hippocampal astrocytes in vitro and in brain slice preparations. Here, TrkB-T1 activation induced ER calcium release and subsequent store-operated calcium entry [21, 140].

## BDNF signaling cascades

**Fig. 3:**
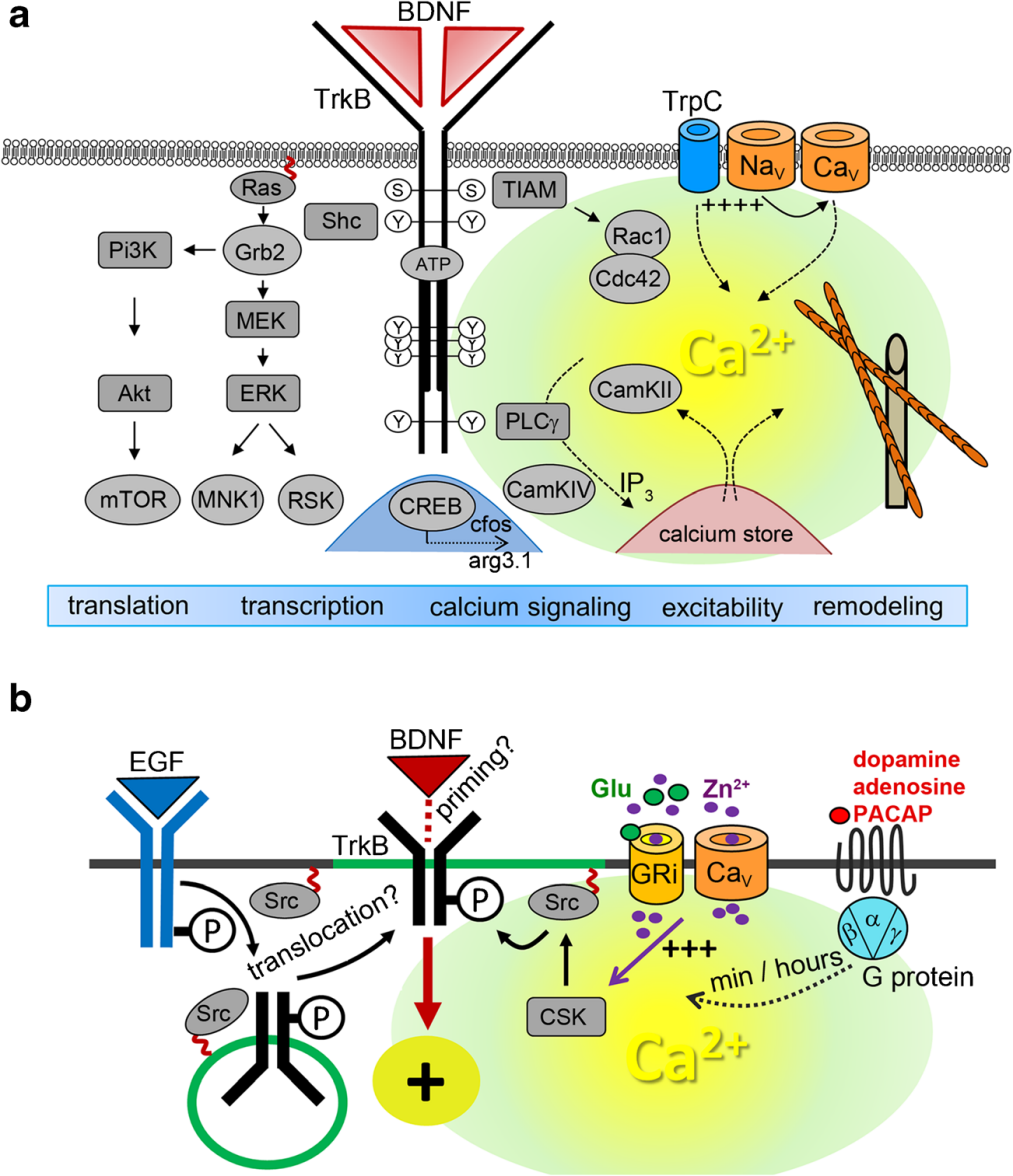
**a** Overview of BDNF/TrkB signaling in neuronal differentiation and synaptic plasticity. **b** Activation of TrkB in the absence of neurotrophins. Details are given in the main text. Abbreviations: *Akt* (protein kinase B), *arg3.1* activity-regulated gene 3.1 protein homolog (Arc), *BDNF* brain-derived neurotrophic factor, *CamK* Ca^2+^/calmodulin-dependent protein kinase, *Ca*_*V*_ voltage-gated calcium channel, *Cdc42* GTPase cell division control protein 42, *cfos* transcription factor cFos, *CREB* transcription factor cAMP response element-binding protein, *CSK* C-terminal Src kinase, *ERK* extracellular signal regulated kinase, *Grb2* growth factor receptor bound protein 2, *GRi* ionotropic glutamate receptors, *IP*_*3*_ inositol 1,4,5-trisphosphate, *MEK* mitogen-activated protein kinase kinase, *MNK* mitogen-activated protein kinase-interacting kinase, *mTOR* mechanistic target of rapamycin, *Na*_*V*_ voltage-gated sodium channel, *PACAP* pituitary adenylate cyclase-activating peptide, *Pi3K* phosphatidylinositol 3-kinase, *PLC* phospholipase C, *Rac* GTPase Ras-related C3 botulinum toxin substrate, *Ras* GTPase rat sarcoma, *RSK* ribosomal S6 kinase, *Shc* Src homologous and collagen-like protein, *Src* Src family of protein tyrosine kinases (SFKs, e.g., Fyn), *TIAM* T cell lymphoma invasion and metastasis-inducing protein, *TrkB* tropomyosin-receptor-kinase B, *TrpC* canonical transient receptor potential channel, *Zn* ^*2+*^ zinc ions

BDNF/TrkB kinase signaling can be divided in (1) signaling cascades which occur over minutes to hours and (2) fast BDNF-induced signaling cascades which excite neurons (outline and abbreviations are found in Fig. 3). BDNF actions differ markedly depending on how fast BDNF concentrations rise when it is delivered to neurons [70, 71, 82].

Upon binding BDNF, TrkB dimerizes, activates an intrinsic kinase activity, undergoes autophosphorylation, and activates a complex set of intracellular signaling cascades [30, 65, 110, 123, 126] (Fig. 3a). The Shc adaptor protein links the activated Trk receptor at Tyr^515^ to the Pi3K/Akt pathway. Activation of Shc by Trk increases in the activity of the small GTPase Ras and the protein kinase ERK (also called MAPK, mitogen-activated protein kinase). Downstream of ERK and Pi3K/Akt, MAP kinase-interacting kinases (MNK), and mTOR-signaling mediates BDNF/TrkB functions for translational control [123, 124] (Fig. 3a).

Phosphorylation of a tyrosine at the carboxyterminal end of TrkB (Tyr^816^) creates the binding site for the PLCγ which subsequently induces the release of calcium ions from the intracellular calcium store (Fig. 3a). Calcium release from internal calcium stores links BDNF signaling with many calcium-dependent signaling steps, e.g., via activation of CaMKII, a master regulator of synaptic plasticity. Phosphorylation of Ser^478^ in the juxtamembrane region of TrkB by CDK5 (cyclin-dependent kinase 5) links TIAM1, a guanine nucleotide exchange factor, to activate Rac1 (Fig. 3a). Rac1 is a well-established signaling protein that promotes spine growth and maturation by regulating actin dynamics [81]. This signaling cascade is involved in BDNF- and activity-dependent dendritic spine remodeling.

BDNF-dependent TrkB activation can immediately excite neurons. BDNF can, for instance, induce a slow and sustained calcium influx and a long-lasting non-selective cationic current through canonical transient receptor potential channels such as TrpC3. This effect is initiated by TrkB-PLCγ activation and is thought to depend on calcium release from internal calcium stores [5, 91–93]. Very fast signaling cascades of BDNF are not well defined [21]. When locally applied, BDNF can also mediate fast sodium influx which triggers fast calcium ion influx through voltage-gated calcium channels [20, 71, 79, 82] (Fig. 3a).

## TrkB activation in the absence of neurotrophins

Activation of TrkB in absence of its natural ligand BDNF, the so-called TrkB transactivation, is another important mechanism how TrkB can exert specific signaling functions [30, 67, 133] (Fig. 3b). Structural models for the activation mechanism of TrkB are mainly based on generalized models of receptor tyrosine kinase (RTK) activation [88]. In general, growth factor binding activates RTKs by inducing receptor dimerization and structural data show that dimerization of the extracellular receptor domain of Trk receptors is mediated by the ligand dimer [158]. In the absence of a ligand, RTKs are autoinhibited in cis and autoinhibition is released following ligand-induced receptor dimerization [88]. However, many studies showed that TrkB can execute autophosphorylation activity and downstream signaling without stimulation by the ligand BDNF [66, 85, 117, 133–135, 159]. Activation of TrkB receptors in the absence of neurotrophins can be mediated by ligand activation of the G-protein-coupled adenosine 2A receptor (A2A-R) or the dopamine D1 receptor [67, 85, 134, 159] (Fig. 3b). G-protein-coupled receptors can trigger the transactivation of TrkB in the range of tens of minutes to hours. This effect is mediated by Src family of protein tyrosine kinases (SFKs, e.g., Fyn) [67, 85, 134, 135]. TrkB activation in the absence of neurotrophins can occur at intracellular sites [67, 133, 135], which includes calcium-dependent steps [67], and regulates the cell surface abundance of TrkB [67, 133] (Fig. 3b). During development of the cortex, intracellular TrkB and TrkC can be activated by an Src kinase-dependent pathway induced by EGF binding to the EGF receptor [133] (Fig. 3b). This effect regulates the migration of newborn cortical neurons. In contrast to the slow G-protein-mediated TrkB activation, EGF-mediated transactivation of TrkB occurs in the range of some minutes and regulates TrkB responsiveness to BDNF at the cell surface [133]. In hippocampal neurons, TrkB can be transactivated by zinc ions, which signal through C-terminal Src kinase (CSK) [61, 66, 121] (Fig. 3b).

Before understanding how other factors transactivate TrkB, more efforts are needed to better understand the structural basis of auto-transactivation between TrkB dimers. TrkB autophosphorylation has been proposed to be a sequential cis/transphosphorylation, with a cis component (a kinases’ own active site mediates autophosphorylation) as an initial key step, while the subsequent transphosphorylation step is dependent on the concentration of the intracellular TrkB kinase domain [68]. We do not know much yet, but cis components in Trk kinase activation belong to those observations that support the provocative idea that active TrkB monomers can be of functional relevance.

## BDNF signaling in synaptic plasticity

Long-term potentiation (LTP) and long-term depression (LTD) are prototypical cellular models of synaptic plasticity by which defined types of synaptic stimulation result in a long-lasting increase (potentiation) or decrease (depression) in the strength of synaptic transmission. An increasing number of studies show that structural spine plasticity and synaptic processes such as LTP or LTD are causally linked to memory [58, 115, 166].

Many studies showed that BDNF is secreted during LTP induction and is functionally essential for acute signaling cascades leading to LTP, albeit BDNF is not required for all forms of LTP or mechanisms leading to LTP [23, 45, 46, 76, 171]. Blocking TrkB or scavenging BDNF during and after LTP induction confirmed critical temporal windows required for early and late phases in BDNF-dependent LTP components that begin with the induction of LTP and end in the range of 10 to 60 min after LTP initiation [45, 49, 57, 66, 76, 78, 79, 104, 145, 150, 155, 171, 172]. Furthermore, there is compelling evidence that memory consolidation needs a persistent action of BDNF over longer time windows of up to 24 h [13, 124]. Some review articles worth reading have been focused on BDNF action in synaptic plasticity and early and late phases of BDNF-dependent components of LTP [23, 46, 123].

**Fig. 4:**
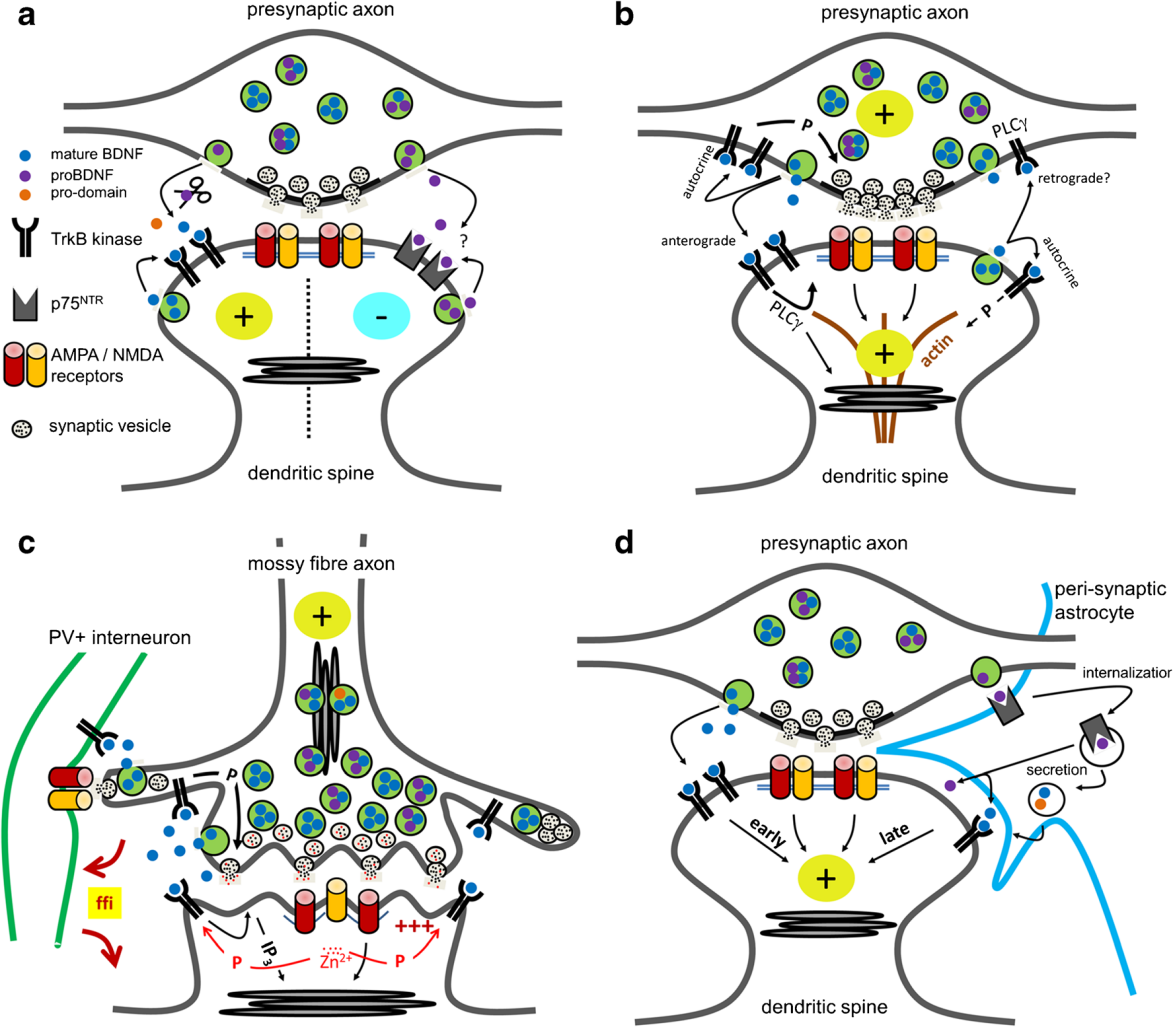
Models of synaptic BDNF signaling. **a** Functional antagonism of BDNF isoforms. In this model, BDNF potentiates the strength of synaptic transmission (*plus sign*) through TrkB receptors, while under different stimulation conditions, postsynaptic p75^NTR^ receptors are activated by proBDNF to decrease the strength of synaptic transmission (*minus sign*). Mature BDNF is either secreted or formed by extracellular cleavage from proBDNF, as indicated by the enzymatic scissor. BDNF may be secreted from pre- and/or postsynaptic sources. **b** Autocrine and paracrine BDNF signaling to increase the strength of synaptic transmission. Presynaptic and postsynaptic TrkB receptors are activated by mature BDNF. TrkB-PLCγ signaling mediates fast BDNF functions and interacts with other phosphorylation cascades to mediate temporally delayed effects, such as structural LTP. BDNF derives from presynaptic and postsynaptic sources and acts autocrine and anterograde. Under certain circumstances, BDNF might act as a potent retrograde messenger. The model includes that TrkB signaling cascades activate synaptic ER calcium stores. The model combines converging lines of evidence at the Schaffer collateral–CA1 synapse. **c** BDNF signaling at the mossy fiber synapse. This model is focused on anterograde BDNF release. High amounts of BDNF are stored in the presynaptic mossy fiber terminal. Cleaved and uncleaved BDNF isoforms are found in this synapse. Autocrine activation of presynaptic TrkB receptors is involved in presynaptic LTP. BDNF can also excite CA3 pyramidal neurons, either through IP_3_-mediated ER calcium release and subsequent activation of canonical transient receptor potential (TrpC) channels or fast depolarization (*triple plus signs in red*). Zinc ions (Zn^2+^) are co-released with glutamate from mossy fiber synapses, enter CA3 pyramidal neurons through ion channels, and mediate postsynaptic TrkB transactivation. BDNF signaling mechanisms at this synapse might change during development. There is evidence that BDNF plays an important role in feedforward signaling through local interneurons. The temporal and spatial aspects of the indicated signaling mechanisms are unclear. **d** BDNF signaling at the tripartite synapse in the perirhinal cortex. Secreted proBDNF is taken up by peri-synaptic glial cells through p75^NTR^ and is internalized and recycled as mature BDNF for LTP maintenance. The locus of proBDNF processing is not known

BDNF in LTP formation is best understood for the hippocampus, but the effects of BDNF differ quite widely depending on the type of synapse. Therefore, it is not well known yet which BDNF signaling cascades contribute to circuit functions in memory processing (Figs. 3 and 4).

### BDNF and LTP formation in the hippocampal CA1 region

A functional role of endogenous BDNF in LTP expression in CA1 was first described by Korte et al. [76]. In hippocampal slices of homo- and heterozygous BDNF knockout mice, LTP expression was reduced after tetanic stimulation [76]. This experiment indicated an immediate effect of BDNF on synaptic plasticity. In another study, it was shown that application of exogenous BDNF for 30 min was sufficient to reverse deficits in LTP and synaptic transmission at the Schaffer collateral–CA1 synapse of 2- to 3-week old BDNF knockout mice. At his age, BDNF knockout mice are old enough to develop LTP, but are still viable [130]. BDNF does not only exert an acute action on CA1 neurons but also triggers synapse maturation. In hippocampal slices of the developing hippocampus, at postnatal day 12–13, different types of tetanic stimulation caused short-term potentiation but did not develop LTP [49]. However, when treated with exogenous BDNF for 2.5–4 h, the same stimulation protocols could induce LTP. Thus, in the immature hippocampus, when corresponding slice preparations are not capable of producing LTP, BDNF exerts a modulatory function to enable an enhanced responsiveness of synapses to plasticity-inducing stimulation protocols. However, in older animals, BDNF can exert an instructive effect during synaptic potentiation. When a function-blocking antibody against BDNF was acutely available 2 min before and 2 min after LTP induction, synaptic potentiation was markedly reduced in hippocampal slices of 4–6-week old mice [78]. One source of BDNF release that is required for LTP induction at CA3-CA1 excitatory synapses is the presynaptic CA3 neuron via its Schaffer collateral [171]. Removing BDNF selectively from CA1 neurons affected neither theta-burst-induced LTP nor high-frequency-dependent LTP (200 Hz LTP). In these experiments, both forms of LTP depended on BDNF in the presynaptic CA3 neuron. Notably, this presynaptic BDNF was shown to regulate a presynaptic module of LTP, possibly via autocrine activation of presynaptic TrkB kinases [171] (Fig. 4b).

Endogenous BDNF can also be triggered by spike-timing protocols, for instance, by combining local glutamate uncaging at spines paired with postsynaptic spikes [57, 60, 150] or by repeated sequential action potential firing in pre- and postsynaptic neurons [45, 46]. BDNF secretion induced by spike-timing protocols can induce a functional timing-dependent LTP (t-LTP), as recently shown for CA1 pyramidal neurons at an age between P15–23 in rats and P25–P35 in mice [45]. In this experimental paradigm, repeated pairing of one presynaptic action potential with four postsynaptic spikes induced a postsynaptic t-LTP. This t-LTP formation was blocked by an acutely applied BDNF scavenging antibody and was attributed to postsynaptic secretion of BDNF by the CA1 neuron itself [45]. This confirms that BDNF is secreted during a protocol for spike-timing-dependent plasticity, a cellular model thought to represent memory formation at the single-cell level [55]. Structural and functional plasticity includes autocrine action of BDNF on TrkB receptors in spines of CA1 pyramidal neurons [57, 60] (Fig. 4b). When local and repetitive glutamate uncaging was performed at individual spines of organotypic slice cultures, calcium influx through NMDA receptors and subsequent activation of postsynaptic CaMKII could trigger the release of postsynaptically synthesized BDNF to induce spine enlargement and LTP [57]. Another set of experiments showed that spine enlargement by autocrine BDNF includes signaling through Rac1 and Cdc42, two small GTP-binding proteins involved in actin cytoskeleton remodeling [60] (outlined in Fig. 4b).

In summary, after developmental maturation of the hippocampus, pre- and postsynaptic TrkB and pre- and postsynaptic BDNF contribute to effects in synaptic plasticity (Fig. 4a, b). It is possible that this complexity reflects a fine tuning mechanism which allows the synapse to rapidly adapt to a new memory situation. Spine turnover rates are much higher in CA1 than in the neocortex, and therefore, it was assumed that the transience of hippocampal-dependent memory is mirrored in the high turnover dynamics of hippocampal synapses [10]. It might well be that BDNF, proBDNF, and the prodomain of BDNF are master regulators of synapse turnover plasticity (Fig. 4a) and that some kind of local BDNF cloud, which covers both the pre- and postsynaptic part of the synapse (Fig. 4b), is needed to produce more stable synaptic connections.

Mice lacking the TrkB receptor for BDNF and mice with a targeted mutation in the PLCγ site of TrkB show LTP deficits as well [110–112]. Notably, concurrent pre- and postsynaptic interference with TrkB-PLCγ signaling was needed to efficiently reduce LTP [51], which is in accordance with pre- and postsynaptic effects of BDNF and TrkB in CA1 LTP (Fig. 4b). TrkB mediates its downstream signaling function through PLCγ activation [111, 114] (Fig. 3a), and it is believed that subsequent calcium release from the endoplasmic reticulum or spine apparatus is important to transduce the signal. However, not all spines carry ER structures or a spine apparatus [148]. The cell biology of presynaptic and postsynaptic calcium stores, which are activated by TrkB-PLCγ signaling, is largely unknown (Figs. 3a and 4b).

### BDNF and LTP formation in the dentate gyrus

A different form of BDNF-mediated plasticity was found in dendrites of dentate granule cells in the hippocampus. Based on the finding that exogenous BDNF, when locally applied for some milliseconds, can exert a fast, neurotransmitter-like action on multiple types of neurons [20, 71, 140], it was asked whether this fast excitatory action of BDNF supports LTP formation. Stimulation of afferents of the medial perforant path combined with a fast and local pulse application of BDNF was sufficient to induce LTP [79]. This LTP required activation of a postsynaptic voltage-gated calcium channels and NMDA receptors. Rapid calcium signals induced by BDNF were exclusively observed in dendritic spines, indicating that postsynaptic spines were the principle site of fast BDNF actions in this form of synaptic plasticity [79]. In vivo, induction of BDNF-LTP, in contrast to HFS-LTP, does not require NMDA receptor (NMDAR) activation, while both BDNF-LTP and HFS-LTP are associated with enhanced granule cell excitability and enhanced synaptic transmission [106]. Notably, in the dentate gyrus, BDNF does not only act as an instructive factor in LTP but also mediates the consolidation of LTP [105, 106, 123, 124]. BDNF activation of TrkB can be very fast, and phosphatases can shut off TrkB function in the range of minutes. However, here in the dentate gyrus, LTP consolidation requires a sustained BDNF/TrkB signaling process. When high frequency stimulation of the medial perforant path was performed in anesthetized rats to induce dentate gyrus LTP, BDNF-dependent elements of LTP lasted for many hours [124]. Notably, rapid, complete, and permanent reversion of LTP could be achieved by local application of a BDNF scavenger at 10 min and 2 or 4 h after LTP induction [124]. The BDNF-dependent phase ended at about 10 h after LTP induction. This persistent BDNF/TrkB signaling process depends on MAP kinase-interacting kinase signaling cascades (Fig. 3a) and activates two mechanistically distinct forms of protein translation. The early phase of BDNF-dependent protein translation occurs in a time window of about 100 min after LTP induction, while the second subsequent late consolidation phase depends on dendritic protein synthesis and lasts hours [23, 105, 106, 123, 124].

### BDNF and LTP formation in CA3 region

The granule neurons of the dentate gyrus provide a strong input to the hippocampus. Furthermore, in the subgranular zone of the dentate gyrus, granule neurons are continuously generated and are functionally integrated into the hippocampal circuit. This projection is critically involved in the processing of contextual elements of memory, memory precision, pattern separation, and/or memory resolution [73, 141]. Notably, highest amounts of BDNF in the entire adult brain are found within the glutamatergic mossy fiber boutons (MFB) [37, 38, 43] (Fig. 2h–j). MFBs form a strong, soma-near, dendritic excitatory contact with thorny spines on CA3 pyramidal neurons, and each MFB forms varicosities and highly dynamic small filopodial extensions to excite GABAergic interneurons [1] (Fig. 4c). These filopodia show experience- and learning-dependent structural plasticity and regulate feedforward inhibition, which is crucial for memory precision [141]. Within the MFBs, BDNF is not only contacting CA3 pyramidal neurons but is also found in mossy fiber terminals contacting inhibitory neurons [38] (Fig. 4c). Mossy fiber-mediated LTP is unusual because its expression is independent of postsynaptic NMDA receptors and presynaptic (non-associative) LTP, albeit mossy fiber synapses evoke NMDA receptor currents [119]. In heterozygous BDNF knockout mice, MF-LTP is drastically reduced [145]. Acute blockade of BDNF/TrkB signaling during MF-LTP induction reduced MF-LTP as well [145]. As BDNF is not found in dendrites of CA3 pyramidal neurons of adult mice [43], and MF-LTP is presynaptic, these experiments indicate that mossy fiber BDNF might act preferentially in an autocrine way on presynaptic TrkB receptors to enhance neurotransmitter release (Fig. 4c). However, CA3 pyramidal neurons express high numbers of the BDNF receptor TrkB, thus raising the question whether there is a role of the postsynaptic TrkB in synaptic plasticity. Surprisingly, epileptogenesis-induced TrkB activation was found in animals with a conditional lack of BDNF, so the question was whether a non-BDNF ligand might be able to activate TrkB. In search of such a ligand, Huang et al. [66] found that the divalent cation zinc, released by mossy fiber terminals, can induce an activity-dependent transactivation of TrkB (Fig. 3b and 4c). This transactivation process is able to potentiate the mossy fiber-CA3 synapse. However, genetic elimination of BDNF in zinc transporter 3 knockout mice was needed to reduce in vivo TrkB phosphorylation at the PLCγ interaction site [61]. This suggests that BDNF but not vesicular zinc activates TrkB in hippocampal mossy fiber axons, at least under steady-state conditions [61]. The interplay of many mechanisms might be involved in BDNF-mediated plasticity at the MF synapse (Fig. 4c). Anyhow, the mossy fiber to CA3 synapse is a promising system to find out how local BDNF signaling affects network maturation, synaptic wiring, and learning-dependent synaptic plasticity in a microcircuit. Furthermore, it is a good system to investigate how pre- and postsynaptic calcium stores act downstream of TrkB.

## BDNF and LTP maintenance in perirhinal cortex

Regulation of endogenous BDNF secretion plays a critical role in perirhinal cortex LTP [2]. Here, critical levels of endogenous BDNF released for 8–12 min after TBS are required for LTP maintenance [2]. The way in which BDNF availability is orchestrated might thus contribute to this form of synaptic plasticity. Accordingly, different phases of LTP are sensitive to BDNF signaling in different brain circuits, and previous investigations in hippocampus demonstrated that longer time windows are required for BDNF action on LTP maintenance. Late TBS-LTP in the CA1 region is prevented when TrkB phosphorylation was blocked by NMPP1 from 1 to 40 min post-TBS but is not affected thereafter [99]. Moreover, high-frequency stimulation (HFS)-induced LTP in the CA1 of hippocampal slices can be inhibited by TrkB-Fc in a time frame of 30 to 60 min, but not from 70 to 100 min, after LTP induction [72]. Even longer periods of BDNF action (about 8 h) are necessary in the dentate gyrus [124]. Whether BDNF availability in individual circuits contributes differently to the duration of BDNF-TrkB signaling and LTP maintenance is hotly debated [23, 46, 126].

In the perirhinal cortex, long-lasting LTP requires the activity of peri-synaptic glia [155]. Here, astrocytes can take up synaptic proBDNF using the carrier receptor p75^NTR^ and recycle it as the mature neurotrophin, in a process essential for LTP maintenance and memory retention [155] (Fig. 4d). Glial BDNF recycling occurs in tight temporal conjunction with the LTP-inducing electrical stimulation, and specifically, deleting p75^NTR^ in glial cells from p75-flox mice [174] prevents BDNF recycling [155]. Notably, when p75^NTR^ is removed from glial cells, TrkB phosphorylation of nearby neurons decreases rapidly following TBS but recovers 10 min later. In accord with this evidence, we demonstrated that exogenous BDNF rescues the late-phase LTP deficit in slices from glia-specific p75 knockout mice only if applied for 10 min from TBS, and indeed, later administrations of exogenous BDNF failed to restore LTP maintenance. Thus, a time-sensitive increase in BDNF availability is required for LTP maintenance [2, 32, 137], and BDNF recycling by glial cells (Fig. 4d) can compensate this physiological requirement. Additionally, recycling is required to sustain the size of the activity-dependent releasable pool of functional BDNF in this cortical area. This might be particularly important for LTP maintenance, which requires threshold BDNF levels [76, 77, 130]. Thus, BDNF recycled by glia [155] or by neurons themselves [144, 164] may synergistically regulate synaptic modifications according to synaptic needs.

Astrocytes are not electrically excitable cells. Receiving signals from neurons at the synaptic cleft is the most expected mechanism for these cells to regulate BDNF recycling. This provides a model by which astrocytes expressing receptors sensing transmitter release from active synapses transduce internal signals leading to endocytic BDNF secretion (Fig. 4d). Astrocytes express receptors for many different transmitters and modulators and display calcium signaling in response to their stimulation. This implies that BDNF glial recycling is a highly regulated process, and it suggests that complex activation of transmitter receptors on glial cells controls final neurotrophin availability. Indeed, both glutamate [17] and the extracellular nucleotide ATP [156] regulate endocytic BDNF secretion, at least in cultured cortical astrocytes. At the tripartite synapse, where astrocytes form a third effective component of the synapse [9, 129], glutamate and ATP signaling between neurons and astrocytes might be needed for maintaining sufficient levels of BDNF for specific synaptic requirements. As glial vesicular release of BDNF is regulated by neurotransmitter release-inducing neuronal activity, astrocytes may then couple neuronal network activity to the local synaptic need of the neurotrophin. Thus, neurons may regulate BDNF glial recycling via the release of distinct transmitters, indicating that neuron-astrocyte interaction can play a much more intricate and functional role in information processing coupled to cognitive functions than previously assumed.

## BDNF signaling and associative learning in psychiatric diseases

Studies in animal models have revealed the involvement of BDNF in higher order functions such as learning, memory, cognition, perception and regulation of emotions. Furthermore, in humans, BDNF and TrkB signaling has been associated with a multitude of psychiatric diseases and is therefore widely studied in the context of schizophrenia, autism, depression, addiction, or anxiety disorders [11]. The concept of treating psychiatric diseases by interacting with BDNF signaling has major caveats. Therapeutics mimicking BDNF or therapies that inhibit or potentiate TrkB signaling may lead to uncontrolled growth effects (e.g., axon sprouting, tumor growth, or cancer cell migration), epileptic seizures and excitotoxicity, or unwished stimulation of long-term changes in synaptic plasticity, for instance, in circuits underlying addiction, reward behavior, or fear and anxiety control. A common view on psychiatric diseases is that altered information processing at synapses in corresponding neural circuits correlates with observed changes in behavior. Therefore, a deep understanding of local, spatiotemporal, and synapse-specific BDNF and Trk (B) function is essential before future therapeutic procedures can evolve from the more detailed knowledge of the corresponding signaling cascades.

In recent years, there has been a growing interest in the role of BDNF signaling in fear and anxiety regulation because associative synaptic learning occurring by fear or anxiety conditioning are thought to be a diathesis for the development of anxiety disorders [109]. Of all psychiatric diseases, anxiety disorders show the highest lifetime prevalence [163], and the underlying neural networks are well conserved between mammalian model organisms and humans [101, 125, 153]. Furthermore, clinical genetic studies suggest a strong genetic contribution to the pathogenesis of anxiety disorders [42, 44]. Thus, fear learning and fear extinction paradigms, which stimulate synaptic plasticity in the underlying neural circuits, are thought to be a good research model for anxiety disorders. For instance, cue conditioning characterized by phasic fear can help to identify neural correlates affected in specific phobias, while context conditioning is assumed to be relevant for anxiety disorders characterized by sustained anxiety [39, 83, 101]. In anxiety disorders, therapeutic interventions such as cognitive behavioral or exposure therapy use beneficial strategies of associative synaptic learning, for instance, extinction learning, to reduce fear memories and anxiety behavior [100]. BDNF is one of the best known synaptic molecules which efficiently modify synaptic strength during associative learning, and it can act as a mediator, modulator, or instructor of synaptic plasticity, over a broad time window. BDNF is one of the most inspiring molecules to better understand the disadvantageous synaptic learning underlying the etiology of anxiety disorders and the beneficial synaptic mechanisms underlying extinction learning [96, 113, 147].

## Concluding remarks

There is compelling experimental evidence that BDNF is an essential factor and instructive mediator of functional and structural plasticity in the CNS and is not only offered to synapses by neurons but also by some types of glial cells. BDNF can exert fast and slow effects at synapses and changes its role during microcircuit development and maturation. All *Bdnf* gene products, proBDNF, mature BDNF, and even the isolated proBDNF domain exert functional activity. proBDNF can not only be regarded as a functional antagonist of mature BDNF, as it can be processed in microcircuits to be re-delivered to synapses. One of the most important features of BDNF is that it acts as a local factor, paracrine and autocrine, on both presynaptic and postsynaptic target sites.

On a molecular level, more structural data are needed to explain how TrkB undergoes intermolecular transactivation and activation in the absence of a ligand. Cell biology in neurons can answer the question how anterograde and retrograde trafficking of BDNF and TrkB is regulated and which individual trafficking steps depend on intracellular TrkB activation. There is a broad lack of knowledge on how BDNF and TrkB are transported and localized within cells. In contrast to our well-developed understanding of BDNF effects, the downstream signaling cascades, the balance between fast and slow signaling, and the corresponding mediators, are not well defined. BDNF is released at synapses, and many BDNF effects in synaptic plasticity are thought to be mediated by calcium release from internal calcium stores. However, not all synapses carry ER structures, and the cellular compartments for local calcium release are barely defined. New techniques that allow direct ER calcium imaging in the presence of extracellular calcium [40, 143] might be able to solve how spatiotemporal BDNF-TrkB signaling activates intracellular calcium stores and how the ER-derived membrane trafficking contributes to synaptic plasticity. Monosynaptic tracing tools in combination with genetic recombination, genome editing, or optogenetic methodology can help to identify the contribution of pre-, post-, or recycled BDNF at individual synapses. New developments in super-resolution microscopy will be advantageous to resolve the local structural dynamics at synapses which are associated with BDNF signaling to show, for instance, how BDNF/TrkB cascades interact with actin or microtubule dynamics, how BDNF is secreted and recycled at synapses, and how TrkB cycles between the cell surface and intrasynaptic membranes. It will be exciting and challenging to look again at the fundamental questions of BDNF neurobiology—where, when, and how. A sound neurobiological foundation for the understanding of BDNF functions in synapse-specific signaling is a prerequisite before BDNF-based therapeutics can become safe and clinically relevant.
